# Supplementary material for: The correlation of salivary telomere length and single nucleotide polymorphisms of the ADIPOQ, SIRT1 and FOXO3A genes with lifestyle-related diseases in a Japanese population
Source: PLoS One. 2021 Jan 28;16(1):e0243745. doi: 10.1371/journal.pone.0243745 (PMC7842940; doi:10.1371/journal.pone.0243745)
Supplement: S1 Fig — (DOCX) [file pone.0243745.s001.docx]

**S1 Fig. Salivary and blood relative telomere length. (A)** In three volunteers (A, B and C), relative telomere length (RTL) was determined by using DNA extracted from saliva and blood-derived whole leukocytes. In volunteer A, the ratios of epithelial cells, neutrophils, lymphocytes and monocytes/macrophages in saliva were 17.4 ± 3.1%, 78.3 ± 6.0%, 1.5 ±1.5%, and 2.8 ± 1.9% [mean ± standard deviation (SD) of triplicate experiments], respectively. The data shown represent the mean ± SD of triplicate RTL measurements. Statistical difference was computed using Student’s *t*-test. **(B)** Similarly, in volunteer A, RTL was determined by using DNA extracted from blood-derived neutrophils(Neut), lymphocytes (Ly) and monocytes (Mo). The neutrophil, lymphocyte and monocyte purity ratios were 94.3 ± 0.5%, 92.9 ± 2.1% and 65.0 ± 3.8% (mean ± SD of triplicate experiments), respectively. The data shown represent the mean ± SD of triplicate RTL measurements. Statistical difference was computed as shown in (**A**).
