# Supplementary material for: The correlation of salivary telomere length and single nucleotide polymorphisms of the ADIPOQ, SIRT1 and FOXO3A genes with lifestyle-related diseases in a Japanese population
Source: PLoS One. 2021 Jan 28;16(1):e0243745. doi: 10.1371/journal.pone.0243745 (PMC7842940; doi:10.1371/journal.pone.0243745)
Supplement: S1 Table — (DOCX) [file pone.0243745.s002.docx]

**S1 Table. Participants’ characteristics, relative telomere length and personal medical histories.**

| **Case** | **Age** | **Sex** | **RTL** | **No.** | **SBP** | **DPB** | **PMH** | | | | | |
| --- | --- | --- | --- | --- | --- | --- | --- | --- | --- | --- | --- | --- |
| **no.** | **y.o.** |  |  | **AHM** |  |  | **HT** | **Stroke** | **AMI** | **CKD** | **Cancer** | **Score** |
| **1** | 53 | Man | 1.450 | 1 | 122 | 80 | 1 | 0 | 0 | 0 | 0 | 1 |
| **2** | 73 | Man | 0.929 | 0 | 120 | 70 | 0 | 0 | 0 | 1 | 0 | 1 |
| **3** | 78 | Man | 0.929 | 1 | 128 | 90 | 1 | 0 | 0 | 0 | 1 | 2 |
| **4** | 66 | Man | 1.053 | 0 | 122 | 66 | 0 | 0 | 0 | 0 | 1 | 1 |
| **5** | 77 | Man | 0.852 | 2 | 134 | 80 | 1 | 0 | 0 | 0 | 0 | 1 |
| **6** | 78 | Man | 1.031 | 0 | 110 | 70 | 0 | 0 | 0 | 0 | 0 | 0 |
| **7** | 77 | Man | 1.0189 | 0 | 122 | 64 | 0 | 0 | 0 | 0 | 0 | 0 |
| **8** | 77 | Man | 0.888 | 1 | 114 | 66 | 1 | 0 | 0 | 0 | 0 | 1 |
| **9** | 65 | Man | 0.858 | 0 | 116 | 76 | 0 | 0 | 0 | 0 | 0 | 0 |
| **10** | 76 | Man | 0.973 | 1 | 102 | 70 | 1 | 0 | 0 | 0 | 0 | 1 |
| **11** | 71 | Man | 1.014 | 0 | 106 | 70 | 0 | 0 | 0 | 0 | 0 | 0 |
| **12** | 72 | Man | 0.823 | 1 | 112 | 78 | 1 | 0 | 0 | 0 | 0 | 1 |
| **13** | 68 | Man | 1.073 | 0 | 126 | 88 | 0 | 0 | 0 | 0 | 0 | 0 |
| **14** | 79 | Man | 1.251 | 1 | 112 | 66 | 1 | 0 | 0 | 0 | 0 | 1 |
| **15** | 77 | Man | 0.931 | 2 | 130 | 70 | 1 | 0 | 0 | 0 | 0 | 1 |
| **16** | 72 | Man | 1.023 | 0 | 106 | 60 | 0 | 0 | 0 | 0 | 0 | 0 |
| **17** | 71 | Man | 0.973 | 0 | 134 | 86 | 0 | 0 | 0 | 0 | 0 | 0 |
| **18** | 72 | Man | 1.133 | 0 | 136 | 88 | 0 | 0 | 0 | 0 | 0 | 0 |
| **19** | 71 | Man | 0.913 | 1 | 122 | 66 | 1 | 0 | 0 | 0 | 0 | 1 |
| **20** | 79 | Man | 0.989 | 2 | 144 | 78 | 1 | 0 | 0 | 0 | 0 | 1 |
| **21** | 76 | Man | 0.986 | 1 | 132 | 76 | 1 | 0 | 0 | 0 | 0 | 1 |
| **22** | 71 | Man | 0.802 | 0 | 124 | 80 | 0 | 0 | 0 | 0 | 1 | 1 |
| **23** | 79 | Man | 0.820 | 0 | 114 | 64 | 0 | 0 | 0 | 0 | 0 | 0 |
| **24** | 77 | Man | 0.843 | 1 | 98 | 60 | 1 | 0 | 0 | 0 | 1 | 2 |
| **25** | 74 | Man | 1.007 | 0 | 134 | 74 | 0 | 0 | 0 | 0 | 0 | 0 |
| **26** | 74 | Man | 0.820 | 2 | 152 | 66 | 1 | 0 | 0 | 0 | 0 | 1 |
| **27** | 80 | Man | 0.786 | 0 | 136 | 82 | 0 | 0 | 0 | 0 | 0 | 0 |
| **28** | 73 | Man | 1.157 | 0 | 128 | 78 | 0 | 0 | 0 | 0 | 0 | 0 |
| **29** | 77 | Man | 0.951 | 1 | 94 | 56 | 1 | 0 | 0 | 0 | 0 | 1 |
| **30** | 78 | Man | 1.498 | 0 | 126 | 76 | 0 | 0 | 0 | 0 | 0 | 0 |
| **31** | 70 | Man | 0.890 | 0 | 114 | 78 | 0 | 0 | 0 | 0 | 1 | 1 |
| **32** | 75 | Man | 0.687 | 0 | 124 | 90 | 1 | 0 | 0 | 0 | 0 | 1 |
| **33** | 66 | Man | 1.553 | 0 | 110 | 84 | 0 | 0 | 0 | 0 | 0 | 0 |
| **34** | 69 | Man | 0.752 | 1 | 108 | 74 | 1 | 0 | 0 | 0 | 0 | 1 |
| **35** | 69 | Woman | 1.150 | 0 | 116 | 64 | 0 | 0 | 0 | 0 | 1 | 1 |
| **36** | 59 | Woman | 0.938 | 0 | 146 | 78 | 1 | 0 | 0 | 0 | 1 | 2 |
| **37** | 73 | Woman | 1.003 | 0 | 148 | 100 | 1 | 0 | 0 | 0 | 0 | 1 |
| **38** | 76 | Woman | 0.853 | 2 | 128 | 78 | 1 | 1 | 0 | 0 | 0 | 2 |
| **39** | 62 | Woman | 0.972 | 0 | 136 | 70 | 0 | 0 | 0 | 0 | 0 | 0 |
| **40** | 59 | Woman | 0.913 | 0 | 155 | 86 | 1 | 0 | 0 | 0 | 1 | 1 |
| **41** | 75 | Woman | 0.467 | 2 | 134 | 56 | 1 | 0 | 0 | 0 | 0 | 1 |
| **42** | 74 | Woman | 0.841 | 0 | 126 | 78 | 0 | 0 | 0 | 0 | 1 | 1 |
| **43** | 72 | Woman | 0.990 | 0 | 134 | 60 | 0 | 0 | 0 | 0 | 0 | 0 |
| **44** | 62 | Woman | 1.014 | 0 | 122 | 80 | 0 | 0 | 0 | 0 | 0 | 0 |
| **45** | 73 | Woman | 0.942 | 0 | 140 | 82 | 1 | 0 | 0 | 0 | 0 | 1 |
| **46** | 68 | Woman | 1.178 | 0 | 142 | 90 | 1 | NA | NA | NA | NA | NA |
| **47** | 71 | Woman | 0.954 | 1 | 126 | 70 | 1 | 0 | 0 | 0 | 1 | 2 |
| **48** | 67 | Woman | 0.956 | 0 | 128 | 70 | 0 | 0 | 0 | 0 | 0 | 0 |
| **49** | 63 | Woman | 0.832 | 0 | 128 | 70 | 0 | 0 | 0 | 0 | 0 | 0 |
| **50** | 67 | Woman | 0.992 | 0 | 128 | 78 | 0 | 0 | 0 | 0 | 0 | 0 |
| **51** | 63 | Woman | 0.958 | 0 | 102 | 70 | 0 | 0 | 0 | 0 | 0 | 0 |
| **52** | 78 | Woman | 0.795 | 3 | 136 | 80 | 1 | 0 | 0 | 0 | 0 | 1 |
| **53** | 62 | Woman | 1.014 | 0 | 126 | 76 | 0 | 0 | 0 | 0 | 0 | 0 |
| **54** | 73 | Woman | 1.017 | 2 | 124 | 64 | 1 | 0 | 0 | 0 | 0 | 1 |
| **55** | 64 | Woman | 1.210 | 1 | 113 | 69 | 1 | 0 | 0 | 0 | 0 | 1 |
| **56** | 69 | Woman | 1.061 | 0 | 164 | 86 | 1 | 0 | 0 | 0 | 0 | 1 |
| **57** | 77 | Woman | 0.828 | 0 | 196 | 90 | 1 | 0 | 0 | 0 | 0 | 1 |
| **58** | 69 | Woman | 0.781 | 0 | 118 | 70 | 0 | 0 | 0 | 0 | 0 | 0 |
| **59** | 72 | Woman | 0.863 | 1 | 128 | 76 | 1 | 0 | 0 | 0 | 0 | 1 |
| **60** | 69 | Woman | 1.213 | 0 | 126 | 62 | 0 | 0 | 0 | 0 | 0 | 0 |
| **61** | 41 | Woman | 1.076 | 0 | 126 | 80 | 0 | 0 | 0 | 0 | 0 | 0 |
| **62** | 71 | Woman | 1.096 | 0 | 136 | 68 | 0 | 0 | 0 | 0 | 0 | 0 |
| **63** | 72 | Woman | 1.099 | 1 | 134 | 70 | 1 | 0 | 0 | 0 | 0 | 1 |
| **64** | 72 | Woman | 1.141 | 0 | 124 | 80 | 0 | 0 | 0 | 0 | 0 | 0 |
| **65** | 66 | Woman | 1.528 | 0 | 158 | 70 | 1 | 0 | 0 | 0 | 0 | 1 |
| **66** | 60 | Woman | 1.201 | 0 | 118 | 68 | 0 | 0 | 0 | 0 | 0 | 0 |
| **67** | 76 | Woman | 1.052 | 0 | 172 | 88 | 1 | 0 | 0 | 0 | 0 | 1 |
| **68** | 77 | Woman | 1.072 | 0 | 126 | 86 | 0 | NA | NA | NA | NA | NA |
| **69** | 71 | Woman | 0.827 | 0 | 138 | 88 | 0 | 0 | 0 | 0 | 0 | 0 |
| **70** | 70 | Woman | 0.964 | 0 | 138 | 70 | 0 | 0 | 0 | 0 | 0 | 0 |
| **71** | 54 | Woman | 0.968 | 1 | 140 | 90 | 1 | 0 | 0 | 0 | 0 | 1 |
| **72** | 64 | Woman | 0.983 | 0 | 106 | 66 | 0 | 0 | 0 | 0 | 0 | 0 |
| **73** | 61 | Woman | 1.445 | 0 | 130 | 81 | 0 | 0 | 0 | 0 | 0 | 0 |
| **74** | 59 | Woman | 0.830 | 0 | 135 | 90 | 1 | 0 | 0 | 0 | 0 | 1 |
| **75** | 50 | Woman | 1.211 | 0 | 122 | 86 | 0 | 0 | 0 | 0 | 0 | 0 |
| **76** | 71 | Woman | 1.127 | 1 | 161 | 92 | 1 | 0 | 0 | 0 | 0 | 1 |
| **77** | 66 | Woman | 1.041 | 0 | 130 | 70 | 0 | 0 | 0 | 0 | 1 | 1 |
| **78** | 64 | Woman | 1.062 | 0 | 106 | 70 | 0 | 0 | 0 | 0 | 0 | 0 |
| **79** | 67 | Woman | 0.99 | 1 | 108 | 66 | 1 | 0 | 0 | 0 | 0 | 1 |
| **80** | 72 | Woman | 1.035 | 1 | 136 | 80 | 1 | 0 | 0 | 0 | 1 | 2 |
| **81** | 65 | Woman | 0.856 | 0 | 104 | 64 | 0 | NA | NA | NA | NA | NA |
| **82** | 70 | Woman | 0.898 | 0 | 108 | 74 | 0 | 0 | 0 | 0 | 0 | 0 |
| **83** | 73 | Woman | 0.872 | 1 | 166 | 86 | 1 | 0 | 0 | 0 | 1 | 2 |
| **84** | 70 | Woman | 0.541 | 0 | 154 | 90 | 1 | 0 | 0 | 0 | 0 | 1 |
| **85** | 69 | Woman | 0.713 | 0 | 122 | 82 | 0 | 0 | 0 | 0 | 0 | 0 |
| **86** | 73 | Woman | 0.705 | 1 | 126 | 60 | 1 | 0 | 0 | 0 | 0 | 1 |
| **87** | 75 | Woman | 0.592 | 0 | 114 | 76 | 0 | 0 | 0 | 0 | 0 | 0 |
| **88** | 66 | Woman | 0.703 | 0 | 112 | 80 | 0 | 0 | 0 | 0 | 0 | 0 |
| **89** | 67 | Woman | 0.756 | 1 | 116 | 64 | 1 | 0 | 0 | 0 | 0 | 1 |
| **90** | 82 | Woman | 0.87 | 3 | 153 | 86 | 1 | 0 | 0 | 0 | 0 | 1 |
| **91** | 70 | Woman | 0.887 | 0 | 110 | 64 | 0 | 0 | 0 | 0 | 0 | 0 |
| **92** | 64 | Woman | 0.998 | 0 | 114 | 76 | 0 | 0 | 0 | 0 | 0 | 0 |
| **93** | 76 | Woman | 0.834 | 0 | 118 | 76 | 0 | 0 | 0 | 0 | 0 | 0 |
| **94** | 69 | Woman | 0.8871 | 2 | 122 | 56 | 1 | 0 | 0 | 0 | 0 | 1 |
| **95** | 65 | Woman | 0.919 | 0 | 116 | 86 | 0 | 0 | 0 | 0 | 0 | 0 |
| **96** | 68 | Woman | 0.854 | 0 | 116 | 76 | 0 | 0 | 0 | 0 | 0 | 0 |
| **97** | 72 | Woman | 1.069 | 0 | 122 | 76 | 0 | 0 | 0 | 0 | 0 | 0 |
| **98** | 73 | Woman | 1.144 | 1 | 156 | 94 | 1 | 0 | 0 | 0 | 0 | 1 |
| **99** | 70 | Woman | 0.913 | 0 | 154 | 70 | 1 | 0 | 0 | 0 | 0 | 1 |
| **100** | 57 | Woman | 0.883 | 0 | 88 | 60 | 0 | 0 | 0 | 0 | 0 | 0 |
| **101** | 70 | Woman | 0.839 | 0 | 164 | 90 | 1 | 0 | 0 | 0 | 1 | 2 |
| **102** | 57 | Woman | 0.945 | 0 | 126 | 70 | 0 | 0 | 0 | 0 | 0 | 0 |
| **103** | 57 | Woman | 0.888 | 0 | 100 | 66 | 0 | 0 | 0 | 0 | 0 | 0 |
| **104** | 66 | Woman | 0.947 | 1 | 170 | 86 | 1 | 0 | 0 | 0 | 0 | 1 |
| **105** | 74 | Woman | 1.074 | 0 | 124 | 76 | 0 | 0 | 0 | 0 | 0 | 0 |
| **106** | 73 | Woman | 1.062 | 1 | 156 | 77 | 1 | 0 | 0 | 0 | 1 | 2 |
| **107** | 78 | Woman | 0.998 | 1 | 130 | 84 | 1 | 0 | 0 | 0 | 1 | 2 |
| **108** | 74 | Woman | 0.842 | 1 | 156 | 76 | 1 | 0 | 0 | 0 | 0 | 1 |
| **109** | 61 | Woman | 0.912 | 0 | 106 | 66 | 0 | NA | NA | NA | NA | NA |
| **110** | 65 | Woman | 0.887 | 0 | 108 | 60 | 0 | 0 | 0 | 0 | 0 | 0 |
| **111** | 60 | Woman | 1.165 | 0 | 124 | 82 | 0 | 0 | 0 | 0 | 0 | 0 |
| **112** | 75 | Woman | 0.882 | 0 | 110 | 68 | 0 | NA | NA | NA | NA | NA |
| **113** | 80 | Woman | 1.097 | 1 | 156 | 86 | 1 | 0 | 0 | 0 | 0 | 1 |
| **114** | 68 | Woman | 0.906 | 0 | 120 | 70 | 0 | 0 | 0 | 0 | 0 | 0 |
| **115** | 69 | Woman | 0.898 | 0 | 106 | 70 | 0 | NA | NA | NA | NA | NA |
| **116** | 51 | Woman | 0.917 | 0 | 102 | 64 | 0 | 0 | 0 | 0 | 0 | 0 |
| **117** | 71 | Woman | 1.185 | 0 | 156 | 78 | 1 | 0 | 0 | 0 | 0 | 1 |
| **118** | 56 | Woman | 0.938 | 1 | 124 | 70 | 1 | 0 | 0 | 0 | 0 | 1 |
| **119** | 64 | Woman | 1.291 | 0 | 116 | 78 | 0 | 0 | 0 | 0 | 0 | 0 |
| **120** | 79 | Woman | 1.001 | 1 | 148 | 88 | 1 | 0 | 0 | 0 | 0 | 1 |

Abbreviations: RTL, relative telomere length; no. AHM, total number of types of prescribed antihypertension medication; Age, age (yr); SBP, systolic blood pressure (BP) (mm Hg); DBP, diastolic BP (mm Hg); PMH, personal medical history; HT, hypertension; AMI, acute myocardial infarction; CKD, chronic kidney disease, which has been treated for edema, hyperkalemia, anemia or edema; Score, sum of the number of affected lifestyle-related diseases, as shown in the materials and methods; 0 for lifestyle-related diseases, no; 1 for lifestyle-related diseases, yes; NA, not available.
