# Supplementary material for: The correlation of salivary telomere length and single nucleotide polymorphisms of the ADIPOQ, SIRT1 and FOXO3A genes with lifestyle-related diseases in a Japanese population
Source: PLoS One. 2021 Jan 28;16(1):e0243745. doi: 10.1371/journal.pone.0243745 (PMC7842940; doi:10.1371/journal.pone.0243745)
Supplement: S2 Table — (DOCX) [file pone.0243745.s003.docx]

**S2 Table. Request for personal medical history.**

1. **Request form written in English**

**2015 “Let’s experience body inspection ! ” research request, request for participation in the health course for**

**the next year and a written consent.**

1. Cooperation in the survey

- I will cooperate with the investigation.
- I can’t cooperate with the investigation.

1. Attendance of next year’s health course

- I will attend
- I will not attend

1. If you are currently receiving treatment for any illness, including medication, please fill in the form.

Currently administered therapeutic agents（　　　　　　　　　　 　　　　　　　）

1. Please circle the numbers that apply to your own diseases below. For other diseases, please enter in ( )

as long as there is no problem.

Affected in the past and now healed

Currently affected

Not affected

Hypertension　 0 1 2

Acute myocardial infarction 0 1 2

Stroke 0 1 2

Chronic kidney disease 0 1 2

Cancer　(any malignant tumor) 0 1 2

Other diseases ( )

1. If you receive health checkup from the city and find any abnormal values in the following items, please

answer with ☑ within the range where there is no problem. If possible, please also enter the inspection value.

□ systolic blood pressure ( ), □ diastolic blood pressure ( ), □ HDL-Cholesterol ( ),

□ LDL-Choresterol ( ), □ Total Choresterol ( ),

□ Triglyceride ( ), □ HbA1c ( ), □ Fasting blood glucose ( ),

□ Other

1. Postal card , Address

Tel , Fax

e-mail

Your name　　　　　　　　　　　　　　　　　　seal

, , 2015

Center for University-Wide Education, Saitama Prefectural University, Koshigaya, 343-8540, Saitama

Tel/Fax:0489(73)4796

Email：[murohashi-ikuo@spu.ac.jp](mailto:murohashi-ikuo@spu.ac.jp)

To Ikuo Murohashi, Principal investigator

1. **Request form written in Japanese**

**2015年度「体験しよう！からだの検査」調査研究、継年健康講座参加のお願いと承諾書**

1. 調査のご協力

- 調査研究に協力します。
- 調査研究に協力できません。

1. 次年度の抗加齢講座のご出席

- 出席します。
- 出席しません。

1. 現在飲み薬などを含めた病気の治療を受けておられましたら、ご記入をお願い致します。

治療薬（　 ）

1. ご自身の以下の疾患につきまして当てはまる数字に○をお願い致します。その他の疾患は

( )に差し支えのない範囲で**ご記入下さい**。

過去にかかった

現在かかっている

かかったことがない

**高血圧症　　　 0 1 2**

**急性心筋梗塞 0 1 2**

**脳卒中 0 1 2**

**慢性腎臓病 0 1 2**

**がん　（すべての種類の悪性腫瘍を含む） 0 1 2**

**その他の疾患；**

1. 以下の直近の定期検診項目で**異常値が御座いましたら**、差し支えのない範囲で☑でお答えを

お願い致します。可能でしたら検査値の記入もお願い致します。

□ 最高血圧( )、 □ 最低血圧（　　）、□ HDL(善玉)コレステロール（　　）、

□ LDL(悪玉）コレステロール(　　)、□ 総コレステロール（　　　）、

□ トリグリセリド (中性脂肪)（　　　）、□ グリコヘモグロビン（　　）、

□空腹時血糖（　　）、□ その他

1. 〒 、ご住所

Tel 、Fax

e-mail

ご氏名　　　　　　　　　　　　　　　　　　印

2015年 月 日

〒343-8540埼玉県越谷市三野宮820番地

埼玉県立大学　 共通教育科 研究代表者　室橋郁生

Tel/Fax:0489(73)4796; メール：[murohashi-ikuo@spu.ac.jp](mailto:murohashi-ikuo@spu.ac.jp)

研究責任者　室橋郁生殿
