# Supplementary material for: The correlation of salivary telomere length and single nucleotide polymorphisms of the ADIPOQ, SIRT1 and FOXO3A genes with lifestyle-related diseases in a Japanese population
Source: PLoS One. 2021 Jan 28;16(1):e0243745. doi: 10.1371/journal.pone.0243745 (PMC7842940; doi:10.1371/journal.pone.0243745)
Supplement: S3 Table — (DOCX) [file pone.0243745.s004.docx]

**S3 Table. Association between relative telomere length and LRD-related physiological and anthropometric measurements.**

| **Men** | | | |
| --- | --- | --- | --- |
| **Parameter** | **Relative telomere length** | | ***p* value** |
|  | **short** | **long** |  |
| **Wt** | 60.66±5.659 (17) | 62.406±8.151 (17) | 0.271 |
| **Ht** | 162.06±5.253 (17) | 167.529±6.637 (17) | 0.016 |
| **BF** | 19.929±5.010 (17) | 19.812±5.478 (17) | 0.871 |
| **BMI** | 23.138±2.466 (16) | 22.200±2.340 (17) | 0.472 |
| **BT** | 36.188±0.616 (17) | 35.943±0.799 (17) | 0.3 |
| **PR** | 73.76±14.520 (17) | 65.29±12.633 (17) | 0.093 |
| **SBP** | 120.00±14.071 (17) | 121.88±12.338 (17) | 0.622 |
| **DBP** | 73.29±9.745 (17) | 74.94±8.547 (17) | 0.773 |
| **WC** | 84.382±7.253 (17) | 85.529±8.283 (17) | 0.345 |
| **IMT** | 0.876±0.217 (17) | 0.888±0.328 (17) | 0.643 |
| **ABI** | 1.156±0.109 (17) | 1.166±0.057 (17) | 0.739 |
| **PWV** | 1578.5±210.2 (17) | 1619.7±303.0 (17) | 0.403 |
| **SPO_2_** | 96.7±1.1 (17) | 96.2±1.6 (17) | 0.353 |
| **Women** | | | |
| **Parameter** | **Relative telomere length** | | ***p* value** |
|  | **short** | **long** |  |
| **Wt** | 51.00±7.535 (43) | 52.158±8.440 (43) | 0.518 |
| **Ht** | 152.55±4.108 (43) | 152.793±5.743 (43) | 0.927 |
| **BF** | 29.767±5.320 (43) | 30.865±5.822 (43) | 0.353 |
| **BMI** | 21.831±2.916 (39) | 21.916±2.932 (38) | 0.882 |
| **BT** | 36.037±0.559 (38) | 35.954±0.491 (35) | 0.406 |
| **PR** | 70.81±11.306 (43) | 71.29±11.195 (42) | 0.847 |
| **SBP** | 128.49±22.162 (43) | 132.14±17.027 (43) | 0.282 |
| **DBP** | 74.37±9.916 (43) | 77.09±9.501 (43) | 0.172 |
| **WC** | 82.360±9.715 (43) | 83.488±9.316 (43) | 0.515 |
| **IMT** | 0.770±0.155 (43) | 0.809±0.273 (43) | 0.295 |
| **ABI** | 1.115±0.072 (43) | 1.115±0.062 (43) | 0.967 |
| **PWV** | 1569.4±317.5 (43) | 1578.5±365.4 (43) | 0.703 |
| **SPO_2_** | 96.3±1.5 (38) | 96.5±1.0 (37) | 0.71 |
| **All** | | | |
| **Parameter** | **Relative telomere length** | | ***p* value** |
|  | **short** | **long** |  |
| **Wt** | 53.74±8.267 (60) | 55.062±9.509 (60) | 0.325 |
| **Ht** | 155.25±6.177 (60) | 156.968±8.960 (60) | 0.2 |
| **BF** | 26.980±6.851 (60) | 27.733±7.582 (60) | 0.652 |
| **BMI** | 22.211±2.834 (55) | 22.004±2.744 (55) | 0.807 |
| **BT** | 36.084±0.576 (55) | 35.951±0.597 (52) | 0.198 |
| **PR** | 71.65±12.246 (60) | 69.56±11.837 (59) | 0.348 |
| **SBP** | 126.08±20.450 (60) | 129.23±16.413 (60) | 0.265 |
| **DBP** | 74.07±9.798 (60) | 76.48±9.221 (60) | 0.157 |
| **WC** | 82.933±9.072 (60) | 84.067±9.013 (60) | 0.351 |
| **IMT** | 0.800±0.180 (60) | 0.832±0.289 (60) | 0.273 |
| **ABI** | 1.127±0.085 (60) | 1.130±0.064 (60) | 0.761 |
| **PWV** | 1572.0±289.4 (60) | 1590.1±346.8 (60) | 0.458 |
| **SPO_2_** | 96.4±1.4 (55) | 96.4±1.2 (54) | 0.788 |

For men, women and all participants, lifestyle-related disease (RLD)-related physiological and anthropometric measurements are shown by relative telomere length (RTL) as shown in the materials and methods. Data are expressed as the mean ± standard deviation. In parentheses, the number of participants who completed the measurements was shown. The *p* value was computed using multivariable linear regression analysis to investigate the association between RTL and LRD-related physiological and anthropometric measurements, and was expressed after adjustments for both sex and age. Abbreviations: Wt, weight (kg); Ht, height (cm); BF, body fat (%); BMI, body mass index (kg/m^2^); BT, body temperature (°C); PR, pulse rate (beats/min); SBP, systolic blood pressure (BP) (mm Hg); DBP, diastolic BP (mm Hg); WC, waist circumference (cm); IMT, carotid maximum intima-medial thickness (mm); ABI, ankle brachial index; PWV, brachial-ankle pulse wave velocity (cm/sec); SpO_2_, saturation of peripheral oxygen (%).
