# Supplementary material for: The correlation of salivary telomere length and single nucleotide polymorphisms of the ADIPOQ, SIRT1 and FOXO3A genes with lifestyle-related diseases in a Japanese population
Source: PLoS One. 2021 Jan 28;16(1):e0243745. doi: 10.1371/journal.pone.0243745 (PMC7842940; doi:10.1371/journal.pone.0243745)
Supplement: S4 Table — (DOCX) [file pone.0243745.s005.docx]

**S4 Table. LRD-related physiological and anthropometric measurements, relative telomere length and frequency of hypertension not associated with any of the three SNPs by genotype.**

| **SNPs** | **Sex** | **Genotype** | **Age** | **RTL** | **Wt** | **Ht** | **BF** | **RR** | **PR** | **SBP** | **DBP** | **HT** | **IMT** |
| --- | --- | --- | --- | --- | --- | --- | --- | --- | --- | --- | --- | --- | --- |
|  |  | **no. (%)** | **y. o.** |  |  |  |  |  |  |  |  | **no. (%)** |  |
|  |  | **n=120** | **n=120** | **n=120** | **n=120** | **n=120** | **n=120** | **n=107** | **n=119** | **n=120** | **n=120** | **n=120** | **n=120** |
| ***ADIPOQ*** | **Men** | GG 18 (53) | 75±4 | 0.98±0.18 | 63±4 | 164±6 | 20.3±3.7 | 14.7±3.4 | 70±15 | 120±15 | 73±10 | 12 (67) | 0.95±0.34 |
|  |  | GT 14 (41) | 72±7 | 1.02±0.23 | 61±9 | 166±8 | 19.8±6.3 | 14.6±3.6 | 69±15 | 123±11 | 75±8 | 3 (21) | 0.81±0.16 |
|  |  | TT 2 (6) | 73±3 | 0.85±0.23 | 54±14 | 163±5 | 16.1±10.0 | 14.0±7.1 | 68±6 | 115±13 | 80±14 | 1 (50) | 0.80±0.14 |
|  | **Women** | GG 56 (65) | 68±7 | 0.97±0.18 | 51±8 | 153±5 | 30.4±6.0 | 14.7±3.4 | 72±11 | 130±21 | 76±11 | 24 (43) | 0.79±0.19 |
|  |  | GT 21 (24) | 65±9 | 0.96±0.13 | 54±9 | 153±5 | 30.3±4.8 | 15.2±4.5 | 70±11 | 127±14 | 75±8 | 9 (44) | 0.76±0.17 |
|  |  | TT 9 (11) | 70±5 | 0.95±0.17 | 49±5 | 150±4 | 30.0±5.0 | 14.9±2.9 | 69±12 | 142±18 | 75±9 | 6 (52) | 0.88±0.44 |
|  | **All** | GG 74 (62) | 70±7 | 0.97±0.18 | 54±9 | 156±7 | 27.9±7.0 | 14.7±3.4 | 71±12 | 127±20 | 75±10 | 36 (49) | 0.83±0.24 |
|  |  | GT 35 (29) | 68±7 | 0.98±0.18 | 57±10 | 159±9 | 26.1±7.5 | 14.9±4.1 | 70±13 | 126±13 | 75±8 | 12 (34) | 0.78±0.17 |
|  |  | TT 11 (9) | 70±5 | 0.94±0.17 | 50±7 | 152±7 | 27.5±7.9 | 14.7±3.5 | 69±11 | 137±20 | 76±9 | 7 (64) | 0.86±0.39 |
| ***SIRT1*** | **Men** | AA 3 (9) | 71±4 | 0.98±0.09 | 61±5 | 170±3 | 18.1±3.6 | 13.7±1.5 | 72±17 | 124±9 | 81±6 | 1 (33) | 0.93±0.12 |
|  |  | AG 12 (35) | 75±4 | 0.98±0.23 | 64±6 | 163±7 | 22.0±3.7 | 15.5±3.6 | 71±12 | 125±17 | 74±10 | 8 (67) | 0.98±0.36 |
|  |  | GG 19 (56) | 72±6 | 1.00±0.20 | 60±8 | 165±6 | 18.8±5.8 | 14.2±3.8 | 68±16 | 118±10 | 73±9 | 7 (37) | 0.82±0.22 |
|  | **Women** | AA 7 (8) | 73±2 | 0.85±0.14 | 53±5 | 151±5 | 33.1±2.0 | 13.5±3.0 | 70±14 | 143±31 | 81±6 | 3 (43) | 0.79±0.15 |
|  |  | AG 39 (45) | 68±6 | 0.98±0.17 | 51±8 | 153±4 | 30.0±6.0 | 14.8±3.3 | 73±13 | 130±19 | 76±10 | 19 (49) | 0.79±0.18 |
|  |  | GG 40 (47) | 66±8 | 0.98±0.17 | 52±9 | 153±6 | 30.2±5.6 | 15.1±4.0 | 69±8 | 128±17 | 74±10 | 17 (43) | 0.79±0.27 |
|  | **All** | AA 10 (8) | 73±3 | 0.89±0.14 | 56±6 | 157±10 | 28.6±7.6 | 13.6±2.5 | 70±14 | 138±28 | 81±6 | 4 (40) | 0.83±0.15 |
|  |  | AG 51 (43) | 70±7 | 0.98±0.19 | 54±9 | 155±7 | 28.1±6.4 | 15.0±3.3 | 73±13 | 129±19 | 76±10 | 27 (53) | 0.84±0.24 |
|  |  | GG 59 (49) | 68±8 | 0.98±0.18 | 54±9 | 157±8 | 26.5±7.8 | 14.8±3.9 | 69±11 | 125±16 | 74±9 | 24 (41) | 0.80±0.25 |
| ***FOXO3A*** | **Men** | TT 19 (56) | 72±6 | 0.98±0.21 | 62±7 | 165±6 | 20.3±5.4 | 14.3±4.0 | 69±15 | 121±12 | 74±9 | 9 (47) | 0.86±0.22 |
|  |  | GT 15 (44) | 74±4 | 1.00±0.20 | 61±7 | 164±7 | 19.3±4.9 | 14.9±3.1 | 70±14 | 121±15 | 75±9 | 7 (47) | 0.91±0.33 |
|  | **Women** | TT 50 (58) | 69±7 | 0.95±0.19 | 52±9 | 153±5 | 30.5±5.4 | 14.8±4.0 | 72±12 | 131±20 | 76±10 | 22 (44) | 0.80±0.19 |
|  |  | GT 36 (42) | 67±7 | 0.99±0.15 | 51±7 | 153±5 | 30.1±5.9 | 14.9±2.9 | 70±10 | 129±19 | 76±9 | 17 (47) | 0.78±0.26 |
|  | **All** | TT 69 (58) | 70±7 | 0.96±0.19 | 55±9 | 156±8 | 27.7±7.1 | 14.7±4.0 | 71±13 | 128±19 | 75±10 | 31 (45) | 0.81±0.20 |
|  |  | GT 51 (42) | 69±7 | 0.99±0.16 | 54±8 | 156±8 | 26.9±7.5 | 14.9±2.9 | 70±11 | 127±18 | 75±9 | 24 (47) | 0.82±0.29 |

For men, women and all participants, lifestyle-related disease (LRD)-related physiological and anthropometric measurements, relative telomere length (RTL) and frequency of hypertension (HT) not associated with any of the three single nucleotide polymorphisms are shown by genotype. Data are expressed as the mean ± standard deviation except for genotype and HT, in which matched numbers and frequencies (%) are shown. The *p* value was computed using multivariable linear regression analysis to investigate the association of genotype with LRD-related physiological and anthropometric measurements, RTL and frequency of HT, and was expressed after adjustments for both sex and age. Abbreviations: SNPs, single nucleotide polymorphisms; Age, age (yr); RTL, relative telomere length; Wt, weight (kg); Ht, height (cm); BF, body fat (%); RR, respiratory rate (breaths/min); PR, pulse rate (beats/min); SBP, systolic blood pressure (BP) (mm Hg); DBP, diastolic BP (mm Hg); HT, hypertension; IMT, carotid maximum intima-medial thickness (mm).
