# Supplementary material for: The correlation of salivary telomere length and single nucleotide polymorphisms of the ADIPOQ, SIRT1 and FOXO3A genes with lifestyle-related diseases in a Japanese population
Source: PLoS One. 2021 Jan 28;16(1):e0243745. doi: 10.1371/journal.pone.0243745 (PMC7842940; doi:10.1371/journal.pone.0243745)
Supplement: Attachment — Submitted filename: PO,Rev,22Oct,Response to Reviewers,IM.docx [file pone.0243745.s006.docx]

‘Response to Reviewers’

Hoh Boon-Peng, PhD

Academic Editor

PLOS ONE, and

Two reviewers

Thank you for your kind and accurate guidance. Responses to academic editor and reviewers are as follows.

Hoh Boon-Peng, PhD

Academic Editor

PLOS ONE

First of all, we apologize for making two mistakes and correcting them. The first is the mistake for statistical processing of quantification of personal medical history. Then, we showed all 120 subjects’ data including age, sex, relative telomere length (RTL) and personal medical history in S1 Table. Due to the correction, the contents of Table 1 have been slightly changed. The second is that we noticed the significant correlation between SNPs ADIPOQ rs1501299 and personal medical history in men. Due to the correction, the contents of Table 2 have been slightly changed.

Next, we will answer your questions in order below.

1. We asked American Journal Expert (AJE) for language editing and proofreading. Certificate of the completion of calibration by AJE is attached.
2. We confirmed by using G*Power software (HHU) that 120 samples are appropriate to provide sufficient power of study. In addition, this study was performed in a single institute in one year in Japanese population. This is described in the revised manuscript.
3. In our paper, a total of 120 subjects were analyzed, 34 men and 86 women. The characteristics of the 120 participants were shown in S1 Table. The description of relative telomere length, three SNPs, hypertension was completed in all 120 subjects. The personal medical history was complete in all 34 men and 80 women, a total of 114. The total of five diseases checked by our trained staff during attendance consisted of hypertension, acute myocardial infarction, stroke and chronic kidney disease, which mainly develop based on vascular lesions, and the rest is cancer (any malignant neoplasms). The sum of the number of affected diseases was compared with the relative telomere length.

Answer to Journal requirements

1. We corrected manuscript to meet PLOS ONE’s style requirements, as far as possible.
2. Our manuscript has been corrected by American Journal Experts, and the certificate of the completion of calibration is attached.
3. “Request for personal medical history” in both Japanese and English was added and shown in S2 Table.
4. I will soon ensure ORCiD ID.
5. We deleted all “data not shown”. All relevant data were shown in S1, S3 and S4 Tables.

Answers to the reviewers’ comments.

1. We have rewritten our revised manuscript extensively according to your guidance.
2. In the Materials and Methods, we rewritted statistical analyses properly.
3. All data were added and shown in S1, S3 and S4 tables.
4. We asked American Journal Expert (AJE) for language editing and proofreading. Certificate of the completion of calibration by AJE is attached. In addition, we read the manuscript many times and corrected the mistakes.
5. Answers to review comments.

Reviewer #1

Thank you for your important advice. We think that our manuscript has been greatly improved owing to the proofreading by American Journal Experts. The certificate of the completion of calibration is attached.

Fig 1 has been changed to S1 Fig, as instructed.

For Table 1, the number of subjects is shown for each group, as instructed.

The number of 34 male subjects is not large, but none were excluded. In addition, relative telomere length determination and personal medical history hearing have been completed for all, as shown in S1 Table. We discussed in depth in the Discussion why the frequency of significant differences are higher in men in the present study.

According to your suggestion for Table 2, we changed the letters the p-values at the end refer to bold font

As suggested, “The effect of heritability on TL is presumed to be weaker in the middle-aged to elderly subjects in the present study than in the younger subjects.“ has been added to the Discussion.

We are very sorry for making an inappropriate description in Fig 3. We replaced prevalence with frequency (percentage). We added axis labelling to both A and B. In the frequency [for example 66.7% (6/9)] only one number comes out, and it is impossible to show as mean ± SD. Multivariable linear regression analyses were used to identify statistical significance. As the number of statistical comparisons is too large to describe, only one (p = 0.057) is shown.

Reviewer #2

1) The abstract has been significantly revised, and was divided to introduction, objectives, methods, results and conclusions.
2) Tables 1 and 2 have been improved to the maximum to make them easier to read.
3) We confirmed by using G*Power software (HHU) that 120 samples are appropriate to provide sufficient power of study. In addition, this study was performed at a single facility in one year in a Japanese population. This is described in the revised manuscript. In our paper, a total of 120 subjects were analyzed, 34 men and 86 women. The characteristics of the 120 participants were shown in S1 Table. The description of relative telomere length, three SNPs, hypertension was completed in all 120 subjects. The personal medical history was complete in all 34 men and 80 women, a total of 114. The number of subjects was indicated in Tables 1 and 2, and Tables S3 and S4. We added the description of “The number of subjects in this study was small, 34 men and 86 women, with a total of 120, so the results of the study may be limited.” to the Discussion.
4) We are very grateful for pointing out a very important issue. We deeply apologize for making an inappropriate statement of “independently associated”. This statement in both Abstract and Discussion has been deleted.
5) We measured RTL according to the method by Cawthon (Nucleic Acids Res 2009; 37:e21, reference 23. In our paper). He showed that the correlation of RTL with terminal restriction fragment length measured by Southern blot was strong (R^2^ = 0.844). In some samples, we repeated the measurements of RTL, again in triplicate, on another day. For analysis, however, the results of the first measurement were used for any of the subjects.
6) In our present study, in men, women and men and women in total, clinical characteristics of the study subjects including score for personal medical history were compared across the median or quartiles of RTL by using the methods shown in statistical analyses. Then, we consider that the heading of the Table 1 of “The association of score for personal medical history with relative telomere length” is correct.
6. Do you want your identity to be public for this peer?

**Yes**
