# Supplementary material for: The correlation of salivary telomere length and single nucleotide polymorphisms of the ADIPOQ, SIRT1 and FOXO3A genes with lifestyle-related diseases in a Japanese population
Source: PLoS One. 2021 Jan 28;16(1):e0243745. doi: 10.1371/journal.pone.0243745 (PMC7842940; doi:10.1371/journal.pone.0243745)
Supplement: Attachment — Submitted filename: 2nd Rev, 24 Nov, Response to Reviewers.docx [file pone.0243745.s007.docx]

November 24th, 2020

PONE-D-20-22106R1

The correlation of salivary telomere length and single nucleotide

polymorphisms of the *ADIPOQ*, *SIRT1* and *FOXO3A* genes with lifestyle-related diseases in a

Japanese population

‘Response to Reviewers in the second revision’

Hoh Boon-Peng, PhD

Academic Editor

PLOS ONE, and

Two reviewers

Thank you for your kind and accurate guidance. Responses to academic editor and reviewers in the second revision are as follows.

Hoh Boon-Peng, PhD

Academic Editor

PLOS ONE

First of all, we deeply apologize for the inappropriate descriptions and explanations for the two most important Figures and Tables. We have made the utmost efforts to improve statistical analyses and the two Figures and Tables. Furthermore, we have added a new subtitle ‘Relative telomere length and score for personal medical history’ following subtitle ‘Relative telomere length and LRD-related physiological and anthropometric measurements’. According to the advice by the reviewer 2 in the first revision, we added new Fig 3 to show reproducibility of salivary relative telomere length measurements. We are confident that this improvement will make it easier for the reader to understand the content of the study.

**Answers to the reviewers’ comments.**

2. The conclusions were improved and drawn appropriately based on the data presented.

3. Statistical analyses in the Materials and methods and descriptions for two Figs and Tables were improved.

6. As noted in 3, descriptions of statistical methods have been corrected to make it easier for the readers to understand. In Fig 2 (A) and (B), adjusted p values for men, women and all participants were added, in addition to the unadjusted p value for men in Fig 2 (B). The heading for Fig 2 has been changed to ‘Frequencies of hypertension’ following to the instruction. Furthermore, we have added a new subtitle ‘Relative telomere length and score for personal medical history’ following subtitle ‘Relative telomere length and LRD-related physiological and anthropometric measurements’ to make it much easier for readers to understand.

According to the advice by the reviewer 2 in the first revision, we added new Fig 3 to show reproducibility of salivary relative telomere length measurements.

7. Yes

We would like to express our deepest gratitude for the many guidance we have received to improve our submitting paper.

With best regards,

Ikuo Murohashi, MD
